# Acute Rabbit Eye Model for Testing Subretinal Prostheses

**DOI:** 10.1167/tvst.8.5.20

**Published:** 2019-10-02

**Authors:** Ying Xiao, Yuqin Wang, Fangting Li, Tiezhu Lin, Kristyn Huffman, Stephanie Landeros, Brandon Bosse, Yi Jing, Dirk-Uwe Bartsch, Scott Thorogood, William R. Freeman, Lingyun Cheng

**Affiliations:** 1 Department of Ophthalmology, Jacobs Retina Center at Shiley Eye Institute, University of California, San Diego, La Jolla, CA, USA; 2 Nanovision Biosciences, Inc., La Jolla, CA, USA

**Keywords:** retinal prosthesis, rabbit eye model, subretinal implantation, acute visual electrophysiology eye model, longitudinal OCT

## Abstract

**Purpose:**

Subretinal prostheses are a novel technology for restoring useful vision in patients with retinitis pigmentosa or age-related macular degeneration. We characterize the surgical implantation technique and functional time window of an acute rabbit eye model for testing of human subretinal prostheses.

**Methods:**

Retinal prostheses were implanted subretinally in 26 rabbits using a two-step technique. Fundus imaging, fluorescein fundus angiography, and optical coherence topography (OCT) were conducted postoperatively from days 1 to 21 to monitor prosthesis positioning and retinal anatomic changes.

**Results:**

Successful implantation and excellent retina apposition were achieved in 84.6% of the rabbits. OCTs showed the overlying retina at full thickness for the first 2 days after implantation. Histology confirmed intact inner layers of the overlying retina until day 3. Progressive atrophy of the overlying retina was revealed by repeated OCTs; approximately 40% of the retina thickness remained on postoperative days 5 and 6.

**Conclusions:**

The two-step implantation technique works well for the rabbit eye model with human prostheses. Rabbit retina may be used for acute electrophysiologic testing of a retinal prosthesis, but is unsuitable for chronic studies due to the merangiotic retina and its limited time window of validity.

**Translational Relevance:**

The improved efficacy in prosthesis surgery using this technique will circumvent the challenges in animal models that provide human-like features critical for the transition into human clinical trials.

## Introduction

Inherited retinal diseases, such as retinitis pigmentosa (RP) and outer retinal dystrophies, including age-related macular degeneration (AMD), afflict millions of individuals causing profound vision impairment.^1^,^2^ To date, several high-profile medical interventions have been tested to rescue the dying photoreceptors associated with these diseases, including retinal pigment epithelium (RPE) transplantation^3^ and various gene therapies.^4^ Even with these interventions, degenerative retinal diseases still eventually lead to blindness in many patients. With rapid development in the bioengineering field, retinal prostheses have become an emerging technology with the potential to restore vision. These retinal prostheses bypass damaged photoreceptor cells and evoke the downstream visual pathway to restore signals to the visual cortex.^5^,^6^

At present, several variations of retinal prostheses are under investigation and some are in clinical trials. Each variation differs in several ways, including material composition, number and spacing of electrodes, return electrode configuration, mechanism of light transduction to electrical stimulation, and implantation site. All devices can be categorized broadly based on the surgical implantation position with respect to the retina as either epiretinal,^7^,^8^ subretinal,^9^–^12^ or suprachoroidal^13^,^14^ implants. While all retinal prosthesis implantations are complex surgeries, compared to subretinal implantation, epiretinal or suprachoroidal devices benefit by being a relatively easier surgical procedure in the rabbit eye model as the retina is much thinner and more fragile than human retina. However, epiretinal implants may cause the streaked phosphenes due to inadvertent activation of the underlying nerve fiber layer while suprachoroidal implants have the choroid layer to limit the minimum distance between stimulating electrodes and target neurons, resulting in a higher charge injection requirement and reduced theoretic resolution. Subretinal implants are inserted in the subretinal space where the degenerated photoreceptors are located. Subretinal approaches have the advantage of being directly adjacent to their target neurons and, therefore, can take advantage of the remaining natural processing circuitry with electrical stimulation. In the development and testing of various retinal prostheses, animal eye models have had a critical role to optimize devices. Rodent eyes have been used as preliminary models for testing and improving devices; however, large animal eyes often are needed in more advanced stages of the investigation. Rabbits are easy to handle and their eye size is much closer to that of a human as opposed to rodent eyes. The major drawback of the rabbit model is the relatively thin and largely avascular retina, rendering subretinal surgery very difficult. However, the rabbit's large eye coupled with low cost and ease of handling is favorable, especially for the early stages of device testing and optimization. We report an optimized two-step surgical procedure (transscleral implantation and pars plana vitrectomy) surgical technique to implant a subretinal prosthetic device designed for the human eye into the rabbit eye.

## Materials and Methods

### Subretinal Prosthetic Device

**Figure 1:**
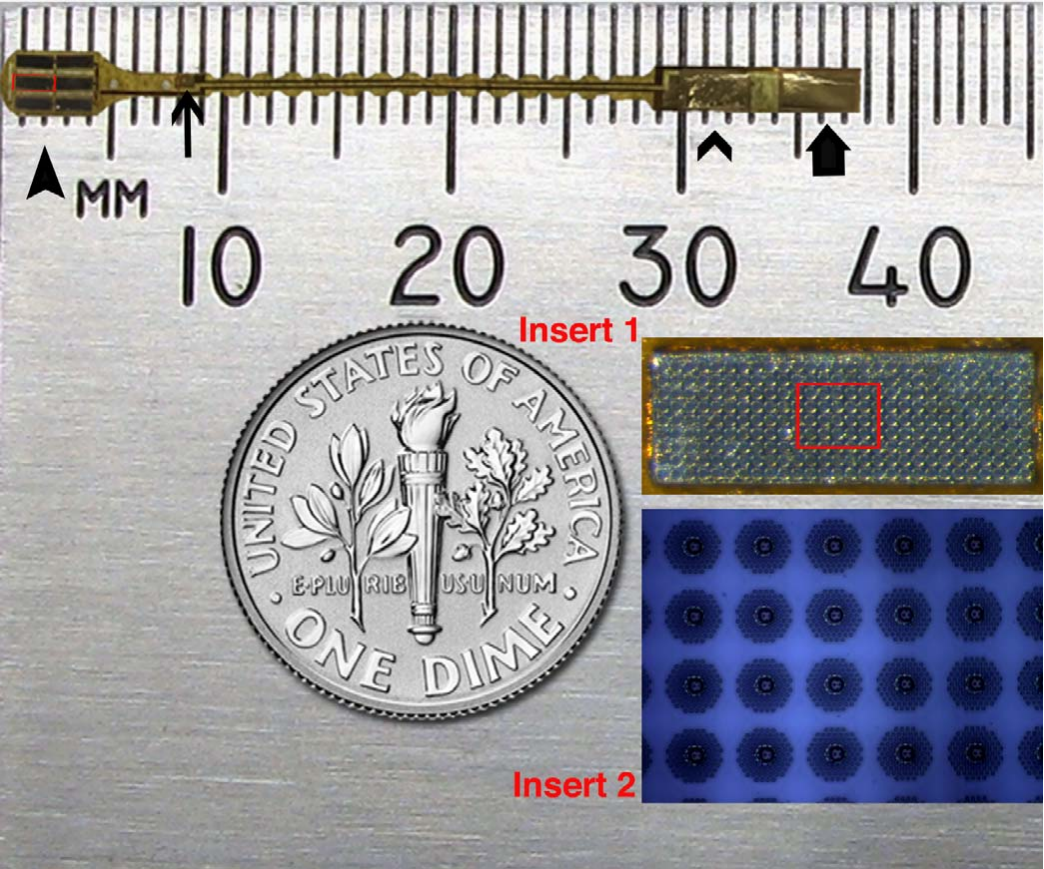
Dimensions of the subretinal prosthetic device. Six silicon chips are arranged on a polyimide substrate at the left end of the device (thick arrowhead). The return electrode is 6 mm away (thin arrow). At the right end of the device, there are two gold-coated electrodes (thin arrowhead pointing to the positive electrode connecting to the chips and the thick arrow indicates the negative electrode connecting to the return electrode). Insert 1 is the magnified view of one chip (red rectangle) and insert 2 is the further magnified view of the red square within insert 1.

The neurostimulation array of the prosthetic device used in our study was comprised of 1512 photovoltaic silicon electrodes on a 12.5-μm thick polyimide substrate.^15^ Each stimulating electrode consists of 85 electrically coupled nanowires connected to an iridium oxide electrode measuring 12 μm in diameter. The stimulating electrodes are divided evenly across six silicon chips measuring 1.4 × 0.5 mm with 252 electrodes per chip and spaced with a pitch of 50 μm. The six chips cover an area of approximately 3 × 4 mm with a thickness of 100 μm (Fig. 1). The vertically aligned nanowires serve as a detector to convert light into electrical current converging to an iridium oxide electrode to stimulate the overlying retinal cells, allowing for an optically addressable array, thereby obviating the requirement for individually-addressed electrodes. The return electrode is 6 mm away from the stimulating electrodes and is on a 23-mm long tail that supplies power to the neurostimulating array. The implant was sterilized in a steam autoclave (Tuttnauer, Hauppauge, NY) for 7 minutes at 134°C and 31 PSI before surgical implantation.

### Animals and Anesthesia

A total of 26 healthy adult New Zealand pigmented rabbits were used in this study. Mean body weight was 3.71 ± 0.55 kg. All animal studies were performed in accordance with the ARVO Statement for the Use of Animals in Ophthalmic and Vision Res. and were approved by the institutional animal care and use committee of University of California, San Diego. Rabbits were anesthetized with subcutaneous injections of 35 mg/kg ketamine hydrochloride and 5 mg/kg xylazine. To maintain normal body temperature, rabbits were put on a warm water blanket during the procedure. Respiratory rate, heart rate, and blood oxygen saturation were monitored continuously. To accommodate the surgical time, additional ketamine was given at half of the original dose every 30 to 40 minutes; 2.5 mg/kg xylazine was given every other redosing.

### Surgical Technique: Two-Step Surgical Implantation

For the surgical procedure, the pupil was fully dilated by 2.5% phenylephrine hydrochloride and 1% tropicamide ophthalmic solution. After induction of general and topical eye (0.5% proparacaine) anesthesia, two traction sutures (1-0 silk) were installed around the superior and inferior muscles to position the eye globe. A third traction suture also was placed in the third eyelid for better exposure of the operative field. A conjunctival peritomy was performed from 2 to 5 o'clock followed by radial cuts of the conjunctiva at 2 and 5 o'clock to create a conjunctival flap. The site of the scleral incision, 5 to 6 mm from the limbus, then was marked with a surgical marking pen. The incision was centered at 3 o'clock and was 3 mm wide. A half-thickness scleral incision was created and followed by cauterizing the choroid before full-thickness cut-through. Two polyimide glides, 3 × 35 mm and 25 μm thick, were coated in diluted Goniovisc and used to flank the prosthetic device in a sandwich fashion. The sandwich assembly then was inserted through the scleral incision into the subretinal space. The sandwich was advanced subretinally under the direct view of a surgical microscope until the neurostimulation array reached the visual streak. Subsequently, the top and bottom polyimide glides were removed before suturing the scleral incision with 7-0 sutures (Supplementary Movie S1). Intraocular pressure (IOP) was restored by intravitreal injection of balanced salt solution (BSS) through the pars plana. After confirmation of a tight closure of the scleral incision around the implant tail, a standard three-port vitrectomy was performed. The irrigation cannula (20-gauge, 2.5 mm long, Eagle Labs, Cucamonga, CA) was installed at 10 o'clock via a 20-gauge MVR blade. The sclerotomy ports for the light pipe at 7 o'clock, and the vitrector at 1 o'clock were made with a 25-gauge MVR blade. A 25-gauge pars plana vitrectomy was performed using the Constellation Vision System (Alcon, Ft. Worth, TX). As much vitreous as possible was removed during the vitrectomy. Conventional air-fluid exchange and silicone oil (ADATO SIL-OL 5000, Bausch and Lomb, Rochester, NY) tamponade was performed. IOP and retinal vessel perfusion were monitored as the oil was infused to avoid high IOP. The air trapped in the vitreous cavity was vented through the irrigation port before closing the sclerotomies using 6-0 sutures. The tail of the implant outside of the globe was tucked under the conjunctiva, which was restored with 8-0 absorbable sutures. 0.5% moxifloxacin hydrochloride eye drops and Tobradex ointment were applied postoperatively and once a day for up to 5 days.

### Postoperative Examinations and Monitoring

After implantation, the eye and implant were monitored by slit-lamp, indirect ophthalmoscope, ocular tonometry, fundus imaging, optical coherence tomography (OCT), and fluorescein fundus angiography (FFA). OCT analysis was conducted on days 1, 2, 3, 5, 6, 7, 8, 14, and 21 to assess prosthesis positioning and evaluate the anatomic changes of the overlying retina over time by using the Spectralis high-resolution, spectral domain OCT imaging system (Software version: Heidelberg Eye Explorer 1.9.10.0; Heidelberg Engineering, Inc., Vista, CA, USA). Images were obtained by line scans (6 mm) over each chip horizontally and vertically. High-quality horizontal scanning OCT images for all six chips were imported into the National Institutes of Health (NIH; Bethesda, MD) software ImageJ 1.51 and retina thickness was measured after calibration. Three locations for each chip, on both ends and the middle of the chip, were chosen to measure the overlying neuroretinal thickness. Off-device retinal thickness served as normal control, which was obtained from three locations 700 to 2000 μm away from the temporal chip edge in the same horizontal scanning line. The average retinal thickness over the chips was compared to the off-device retina thickness over time. FFA was performed without anesthesia on the first postoperative day with both eyes fully dilated. The Spectralis imaging system was used for fluorescence angiography after intravenous injection of 0.1 mL 12.5% sodium fluorescein solution.

## Histologic Study

**Figure 2:**
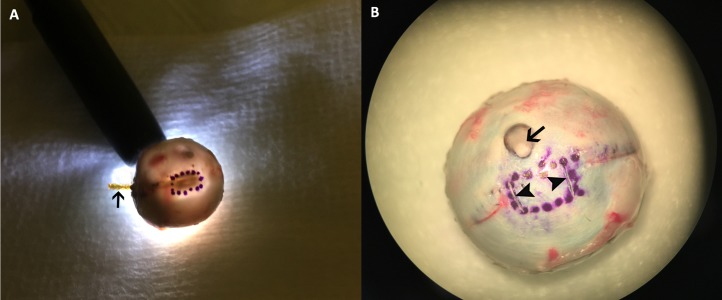
The boundaries of the device head were marked before explanting. The device head is labeled by shining a bright penlight through the pupil in a dimly-lit room. The shadow cast onto the posterior sclera corresponds to the location of the array (A). The head of the device can be seen clearly within the marked circle and the implant tail (arrow) sticks out from the scleral incision. This part of the tail was buried under the conjunctiva at the end of the implantation procedure and used to connect to external electrodes for recording of electrical evoked potential (EEP) before sacrifice. To locate the retinal area of interest (overlying device head) in histologic sectioning, the sclera of the globe was partially cut by a fine needle tip, as shown by two arrowheads in (B). The arrow in (B) indicates the optic nerve head of the rabbit eye globe.

The rabbits were sacrificed after a postoperative period ranging from 1 to 21 days with a lethal dose of sodium pentobarbital (120 mg/kg) under deep anesthesia (16 rabbits on day 1, 3 on day 3, 2 on day 6, 1 on day 14, and 1 on day 21). Nineteen devices were explanted for further engineering tests and analysis. Two eyes at postoperative day 1, one at postoperative day 3, and one at postoperative days 7 and 21 each were processed for histology and light microscopy. The rigidity of the device prevents paraffin sectioning and must be removed, which disturbs and lifts the overlying retina even after the eye globes have been fixed in Davidson's solution. To reduce collateral damage to the retina, transillumination was used to visualize the device head (Fig. 2A) and its boundary was marked on the sclera. Two shallow cuts on the sclera along the temporal and nasal boundaries of the device head were made with a 31-gauge needle to mark the area of retina overlying the device head for identification in the histology sections (Fig. 2B). The subretinal prosthesis was carefully explanted after the above labeling process. For the eyes with longer observation (days 7 and 21), due to substantial thinning and fibrosis of retina overlying the device head, it is impossible to explant the device and leave the overlying retina intact for histology. For these eyes, the implant was not explanted and paraffin sections were taken from immediately next to the chips. All paraffin sections were 5 μm thick and stained by hematoxylin and eosin (H&E) for light microscopy.

### Data Analysis

Retinal thickness at each location was expressed as mean ± standard deviation. The thickness of the retina over the implant and the thickness of the nearby off-device retina were compared at each time point using a paired *t*-test. *P* < 0.05 was considered statistically significant. Statistical analysis was performed with JMP statistical software (JMP, Version <13>. SAS Institute Inc., Cary, NC, 1989–2007).

## Results

### Two-Step Implantation Technique

**Figure 3:**
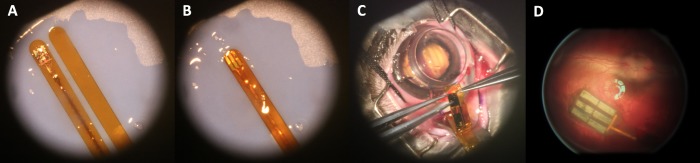
Sandwich assembly (A, B) and two-step surgical implantation (C). Fundus image of the prosthesis in the subretinal space was taken one day postoperatively (D), demonstrating successful surgical implantation of the device under the visual streak in the subretinal space.

Figure 3 demonstrates the assembly of the implant and two polyimide glides into a sandwich structure before surgical insertion, the inserted sandwich assembly, and the successfully implanted device in a rabbit eye. Successful subretinal implantation was achieved in 84.6% of the surgeries (22 of 26 rabbits). Failure reasons included implantation insertion failure (two rabbits) and retinal detachment during vitrectomy from infusion cannula displacement and choroidal hemorrhage (two rabbits). In all 22 rabbits with successful implant surgeries, the prosthetic device was implanted under the visual streak without retinal detachment. Small retinal breaks were sometimes noted along the insertion pathway and were closed after extrusion under air and subsequent tamponade with silicon oil. The retina overlying the array was verified to be intact with postoperative OCT.

### Clinical Examination and Fundus Imaging

**Figure 4:**
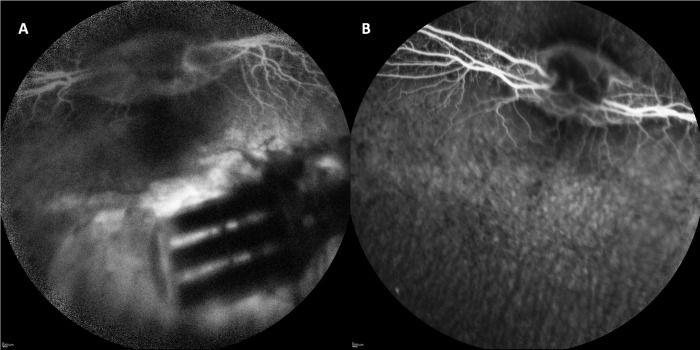
FFA one day after implantation of a subretinal device (A) along with the fellow eye (B). Retinal vessel and background choroidal perfusion were visible, though less clear than the fellow eye due to suboptimal optical media and oil filling. The obstruction of background fluorescence was noted in the areas corresponding to the chips and the substrate of the device while hyperfluorescence was present surrounding the device and between the chips.

Postoperative examinations revealed mild corneal swelling and anterior chamber reaction on postoperative day 1. Limited peripheral lens opacity was found in three rabbits without impeding fundus observation. Indirect ophthalmoscopy revealed the prosthesis in the desired position, which remained stable for all time points. The highly reflective chips were seen through the transparent rabbit retina. Some pigment changes were seen along the insertion pathway where the glides and device were introduced. Due to the unique retinal vasculature of the rabbit, the retina became very thin around day 7 and details of the subretinal array became more visually striking. An atrophic hole in the retina overlying some devices was noted upon fundus examination. FFA on day 1 showed well-demarcated hypofluorescence corresponding to the array and substrate due to the blockage of background fluorescence (Fig. 4A). Near the implant, moderate hyperfluorescence was noted in the pathway where the glide and device were inserted.

FFA also demonstrated less fluorescent retinal vessels on the medullary ray due to hazy optical media and silicon oil filling. Otherwise, no other noticeable findings, such as leakage and partial or total obstruction of vessels, were detected.

**Figure 5:**
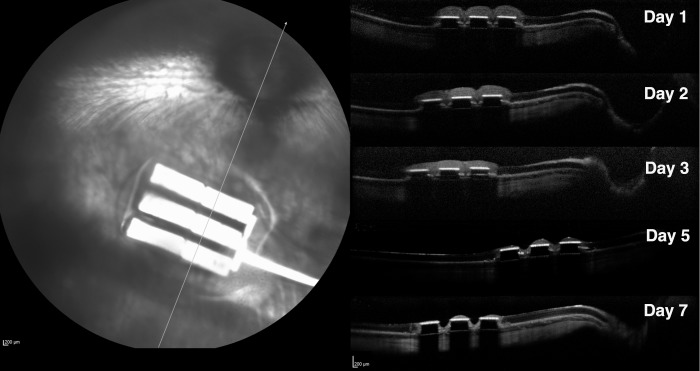
OCT images illustrating retinal changes over time (day 1 to day 7) postoperatively. The three flat reflective bands on the OCT images correspond to the three chips on the neurostimulating array shown in the SLO fundus image. The chips blocked the tissue underneath from being seen on OCT. Retina on the chips in OCT demonstrates thinning from postoperative days 1 to 7.

**Figure 6:**
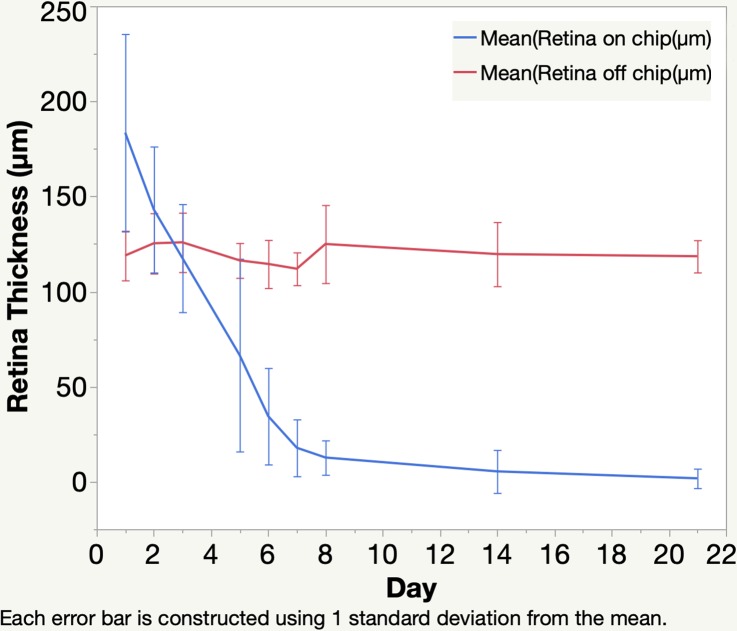
Graph depicting retinal thickness on the chip over time after implant surgery. At postoperative days 1 and 2, the retina was thicker than the nearby retina off the chips. From postoperative day 3 and on, the retina on the chips experienced progressive thinning over time.

OCT demonstrated that the position of the subretinal implants remained stable during the whole observation period. In OCT imaging, highly reflective bands with well-defined demarcation corresponded to the chips. The nontransmissible nature of the chips caused extinction of the OCT beam, which prevented Bruch's membrane and the choroid underneath the implant from being seen. The overlying retina was well attached to the chips and no subretinal fluid was present. The retina overlying the chips on postoperative days 2 and 3 demonstrated less clear layered retinal structures on OCT owing to higher tissue reflectivity. Prominent thinning and even segmental diminishing of the overlying retinal band was observed after day 5, revealing progressive retinal atrophy overlying the prosthesis on OCT (Fig. 5). The average thickness of the normal control retina (off-device) was 119.79$\newcommand{\bialpha}{\boldsymbol{\alpha}}\newcommand{\bibeta}{\boldsymbol{\beta}}\newcommand{\bigamma}{\boldsymbol{\gamma}}\newcommand{\bidelta}{\boldsymbol{\delta}}\newcommand{\bivarepsilon}{\boldsymbol{\varepsilon}}\newcommand{\bizeta}{\boldsymbol{\zeta}}\newcommand{\bieta}{\boldsymbol{\eta}}\newcommand{\bitheta}{\boldsymbol{\theta}}\newcommand{\biiota}{\boldsymbol{\iota}}\newcommand{\bikappa}{\boldsymbol{\kappa}}\newcommand{\bilambda}{\boldsymbol{\lambda}}\newcommand{\bimu}{\boldsymbol{\mu}}\newcommand{\binu}{\boldsymbol{\nu}}\newcommand{\bixi}{\boldsymbol{\xi}}\newcommand{\biomicron}{\boldsymbol{\micron}}\newcommand{\bipi}{\boldsymbol{\pi}}\newcommand{\birho}{\boldsymbol{\rho}}\newcommand{\bisigma}{\boldsymbol{\sigma}}\newcommand{\bitau}{\boldsymbol{\tau}}\newcommand{\biupsilon}{\boldsymbol{\upsilon}}\newcommand{\biphi}{\boldsymbol{\phi}}\newcommand{\bichi}{\boldsymbol{\chi}}\newcommand{\bipsi}{\boldsymbol{\psi}}\newcommand{\biomega}{\boldsymbol{\omega}} \pm 14.31$ μm. The average retinal thickness overlying the chips significantly increased to 183$\newcommand{\bialpha}{\boldsymbol{\alpha}}\newcommand{\bibeta}{\boldsymbol{\beta}}\newcommand{\bigamma}{\boldsymbol{\gamma}}\newcommand{\bidelta}{\boldsymbol{\delta}}\newcommand{\bivarepsilon}{\boldsymbol{\varepsilon}}\newcommand{\bizeta}{\boldsymbol{\zeta}}\newcommand{\bieta}{\boldsymbol{\eta}}\newcommand{\bitheta}{\boldsymbol{\theta}}\newcommand{\biiota}{\boldsymbol{\iota}}\newcommand{\bikappa}{\boldsymbol{\kappa}}\newcommand{\bilambda}{\boldsymbol{\lambda}}\newcommand{\bimu}{\boldsymbol{\mu}}\newcommand{\binu}{\boldsymbol{\nu}}\newcommand{\bixi}{\boldsymbol{\xi}}\newcommand{\biomicron}{\boldsymbol{\micron}}\newcommand{\bipi}{\boldsymbol{\pi}}\newcommand{\birho}{\boldsymbol{\rho}}\newcommand{\bisigma}{\boldsymbol{\sigma}}\newcommand{\bitau}{\boldsymbol{\tau}}\newcommand{\biupsilon}{\boldsymbol{\upsilon}}\newcommand{\biphi}{\boldsymbol{\phi}}\newcommand{\bichi}{\boldsymbol{\chi}}\newcommand{\bipsi}{\boldsymbol{\psi}}\newcommand{\biomega}{\boldsymbol{\omega}}.53 \pm 15.93$ and 143.13$\newcommand{\bialpha}{\boldsymbol{\alpha}}\newcommand{\bibeta}{\boldsymbol{\beta}}\newcommand{\bigamma}{\boldsymbol{\gamma}}\newcommand{\bidelta}{\boldsymbol{\delta}}\newcommand{\bivarepsilon}{\boldsymbol{\varepsilon}}\newcommand{\bizeta}{\boldsymbol{\zeta}}\newcommand{\bieta}{\boldsymbol{\eta}}\newcommand{\bitheta}{\boldsymbol{\theta}}\newcommand{\biiota}{\boldsymbol{\iota}}\newcommand{\bikappa}{\boldsymbol{\kappa}}\newcommand{\bilambda}{\boldsymbol{\lambda}}\newcommand{\bimu}{\boldsymbol{\mu}}\newcommand{\binu}{\boldsymbol{\nu}}\newcommand{\bixi}{\boldsymbol{\xi}}\newcommand{\biomicron}{\boldsymbol{\micron}}\newcommand{\bipi}{\boldsymbol{\pi}}\newcommand{\birho}{\boldsymbol{\rho}}\newcommand{\bisigma}{\boldsymbol{\sigma}}\newcommand{\bitau}{\boldsymbol{\tau}}\newcommand{\biupsilon}{\boldsymbol{\upsilon}}\newcommand{\biphi}{\boldsymbol{\phi}}\newcommand{\bichi}{\boldsymbol{\chi}}\newcommand{\bipsi}{\boldsymbol{\psi}}\newcommand{\biomega}{\boldsymbol{\omega}} \pm \,33.12$ μm on day 1 and day 2, respectively (*P* < 0.0001 and *P* = 0.0004, paired *t*-test). On day 3, retinal thickness decreased to 117.58$\newcommand{\bialpha}{\boldsymbol{\alpha}}\newcommand{\bibeta}{\boldsymbol{\beta}}\newcommand{\bigamma}{\boldsymbol{\gamma}}\newcommand{\bidelta}{\boldsymbol{\delta}}\newcommand{\bivarepsilon}{\boldsymbol{\varepsilon}}\newcommand{\bizeta}{\boldsymbol{\zeta}}\newcommand{\bieta}{\boldsymbol{\eta}}\newcommand{\bitheta}{\boldsymbol{\theta}}\newcommand{\biiota}{\boldsymbol{\iota}}\newcommand{\bikappa}{\boldsymbol{\kappa}}\newcommand{\bilambda}{\boldsymbol{\lambda}}\newcommand{\bimu}{\boldsymbol{\mu}}\newcommand{\binu}{\boldsymbol{\nu}}\newcommand{\bixi}{\boldsymbol{\xi}}\newcommand{\biomicron}{\boldsymbol{\micron}}\newcommand{\bipi}{\boldsymbol{\pi}}\newcommand{\birho}{\boldsymbol{\rho}}\newcommand{\bisigma}{\boldsymbol{\sigma}}\newcommand{\bitau}{\boldsymbol{\tau}}\newcommand{\biupsilon}{\boldsymbol{\upsilon}}\newcommand{\biphi}{\boldsymbol{\phi}}\newcommand{\bichi}{\boldsymbol{\chi}}\newcommand{\bipsi}{\boldsymbol{\psi}}\newcommand{\biomega}{\boldsymbol{\omega}} \pm \,28.39$ μm, which was significantly thinner than the nearby normal retina, 125.86 μm (*P* < 0.0181, paired *t*-test), and progressively dropped to 17.82$\newcommand{\bialpha}{\boldsymbol{\alpha}}\newcommand{\bibeta}{\boldsymbol{\beta}}\newcommand{\bigamma}{\boldsymbol{\gamma}}\newcommand{\bidelta}{\boldsymbol{\delta}}\newcommand{\bivarepsilon}{\boldsymbol{\varepsilon}}\newcommand{\bizeta}{\boldsymbol{\zeta}}\newcommand{\bieta}{\boldsymbol{\eta}}\newcommand{\bitheta}{\boldsymbol{\theta}}\newcommand{\biiota}{\boldsymbol{\iota}}\newcommand{\bikappa}{\boldsymbol{\kappa}}\newcommand{\bilambda}{\boldsymbol{\lambda}}\newcommand{\bimu}{\boldsymbol{\mu}}\newcommand{\binu}{\boldsymbol{\nu}}\newcommand{\bixi}{\boldsymbol{\xi}}\newcommand{\biomicron}{\boldsymbol{\micron}}\newcommand{\bipi}{\boldsymbol{\pi}}\newcommand{\birho}{\boldsymbol{\rho}}\newcommand{\bisigma}{\boldsymbol{\sigma}}\newcommand{\bitau}{\boldsymbol{\tau}}\newcommand{\biupsilon}{\boldsymbol{\upsilon}}\newcommand{\biphi}{\boldsymbol{\phi}}\newcommand{\bichi}{\boldsymbol{\chi}}\newcommand{\bipsi}{\boldsymbol{\psi}}\newcommand{\biomega}{\boldsymbol{\omega}} \pm \,14.95$ μm on day 7. The retina overlying the implant lost 90% of its thickness by day 8 and had almost disappeared by day 14. Thickness decreased to only a few micrometers and was difficult to quantitate accurately on day 21 (Fig. 6). A multivariate regression analysis in a mixed model revealed that the retina overlying the implant became significantly thinner over time (*P* < 0.0001), however, the difference in retinal thickness among the individual chips on the implant was not significantly different (*P* > 0.05).

### Histology Outcome

**Figure 7:**
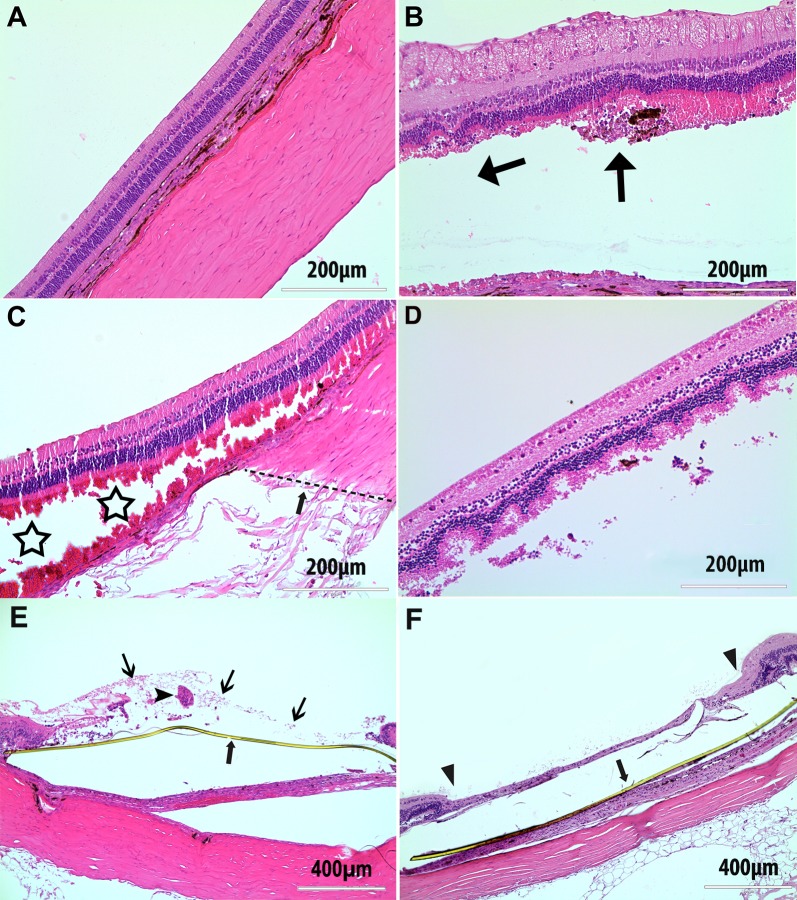
Retinal histology from rabbit eyes 1 (A–C) and 3 (D) days, and 1 (E) and 2 (F) weeks after surgical implantation. (A) Normal retinal structure from the nearby retina with no contact by surgical glides or a prosthetic device. (B) Displays the retina overlying the device head with its outer nuclear layer partially stripped off. The horizontal arrow indicates the implant position (removed) and the vertical arrow indicates the ending point of the microarray head. (C) The retina from a location at the inferior edge of the implant head, as indicated by the dotted line in the sclera (arrow). Stars show splitting of the photoreceptor outer segment by the device head. (D) Marked swelling of cells in the retina, especially in inner nuclear layer, overlying the implant 3 days postoperatively. (E) Autolysis decomposition of overlying (above yellowish polyimide strip, thick arrow) retina on day 7, which lost the layered structure with cells debris (thin arrows) and remnants of degenerated retina (arrowhead). (F) Histology 2 weeks after surgical implantation demonstrating atrophy of the retina overlying the retinal prosthesis device (between two arrowheads) with the yellow polyimide substrate of the device underneath (arrow).

One day after implantation, histology revealed detachment of the retina overlying the implant due to the explanting procedure before histology processing. Adjacent off-device retina was normal (Fig. 7A). The overlying retina had intact retinal structures from the outer plexiform and above (Figs. 7B, 7C). At the site of insertion, the outer nuclear layer was partially stripped off from the implant insertion procedure as seen in Figure 7B. The retina in Figure 7B was close to the optic nerve, thereby appearing thicker due to the myelinated nerve fiber layer than the retina in Figure 7C, which was close to inferior edge of the implant, without myelinated nerve fiber layer (Fig. 7C). The inner retinal layers of the overlying retina in Figures 7B and 7C were relatively well preserved. RPE integrity underneath the device was variable, ranging from obviously damaged (Fig. 7B) to better preserved (Fig. 7C), depending on the location in relation to the implant insertion pathway. The overlying retina on day 3 (Fig. 7D) came from an equivalent location as in figure 7C; however, the retina in Figure 7D demonstrated marked swelling of retinal cells, especially the cells in the inner nuclear layer. On day 7, the overlying retina had lost their layered structure with remnants of autolyzed retinal cells or isolated condensed retinal fibrosis (Fig. 7E). Two weeks after implantation, the retina overlying the implant had no appreciable retina layers; only a thin sheet of gliosis remained over the device (Fig. 7F).

## Discussion

Retinal degenerative diseases mainly involve photoreceptor degeneration as seen in RP and AMD. The inner retina, approximately 40% to 88% of inner nuclear layer cells, and approximately 20% to 30% of ganglion cells survive^16^–^18^ and make it possible to use a subretinal prosthesis to bypass part of the neural circuit and electrically stimulate the remaining retinal neurons to restore useful sight for patients. Engineered prostheses often require a series of incremental engineering optimizations and testing in animal eye models before moving on to clinical trials. Therefore, a dependable and inexpensive eye model for prosthesis optimization is critical to advance the field. Several mammal species have been used in preclinical investigations for bionic eye research, including rodent, rabbit, cat, sheep, dog, and pig.^19^ Among those, rabbits have proven to be the easiest to handle and the most economical, while still providing a relatively large eye size to perform a standard vitrectomy^20^,^21^ and optimization testing. Furthermore, the rabbit's human-like sized eye provides the added benefit of testing human-sized prostheses which then can be smoothly transitioned into human clinical trials. Eye size is an important factor in retinal prosthesis research because the size of the light sensor matters for the engineering and the ocular surgery technique. The main limitation of the rabbit model is its merangiotic retina, which is thin and delicate, posing a challenge in the surgical implantation. We demonstrated a two-step surgical technique to improve the efficacy of implanting a device designed for the human eye in the rabbit eye model. The surgery success rate reached 85% and the implanted rabbit eye was further used to test the visual electrophysiology of the implanted device as shown in our recent study.^22^ We sandwiched the device between two polyimide strips to facilitate insertion of the device into the subretinal space with the use of an appropriate amount of viscoelastic material to reduce friction-related retinal damage. It has been reported that subretinal retention of viscoelastic material over prostheses could be a reason for persistent local retinal detachment after implantation surgeries.^23^ Any fluid accumulation between the device and overlying retina also will increase electrode resistance and decrease neuroresponse to light stimulation. Therefore, our technique avoids the use of Healon or BSS for assisted retinal detachment before insertion.^24^,^25^ Satisfactory reattachment of the overlying retina to the device and surrounding RPE-Bruch's membrane complex was achieved by air–fluid–silicon oil exchange. Good retina apposition to the device is extremely important for efficient signal transduction to the visual cortex. While poor apposition often is seen postimplantation,^26^ in our study the excellent apposition of overlying retina to the device underneath was confirmed postoperatively by OCT.

Several subretinal prosthesis studies confine their electrophysiology exams to same day testing immediately postoperatively; however, it may be advantageous to record electrophysiology beyond this time point.^23^,^25^,^27^–^29^ Long surgical procedures introduce tremendous physiologic trauma to the retina, including light bleaching from the surgical microscope and endoillumination during vitrectomy. Therefore, recording electrophysiology on postoperative days may provide more accurate and reliable data. In our study, spectral domain OCT and retinal fluorescein angiography revealed edematous overlying retina on postoperative days 1 and 2 before subsequent rapid thinning of the overlying retina. The electrophysiology recordings from day 1 postoperatively demonstrated clear full-field and focal VEPs.^22^ The VEPs reported in our previous publication^22^ were similar to the VEPs from same-day studies by Schwahn et al.^27^,^30^,^31^ Noninvasive OCT monitoring of the retina in our study clearly demonstrated rapid morphologic evolution of the overlying retina postoperatively for the first time to our knowledge. The study revealed a short time window of this model for the evaluation of retinal prostheses. Although the retina overlying the implant within the first 3 days is not completely normal due to surgical stress and possible poor nutrient diffusion from the choroid due to blockage from the implant, the eyes demanding a retinal prosthesis have inner retinal abnormalities in addition to the damaged outer retina, such as eyes with RP or AMD. Therefore, the recorded signals from an abnormal overlying retina in the current study still may be valuable to drive implant engineering optimization. After postoperative day 3, the retina overlying the implant experienced a fast thinning and may not be functional enough for acute testing of retinal electrophysiology. For longer-term testing, an animal model, such as canine or swine, with holangiotic retina is necessary. While other studies with rabbits also have used OCT to image the overlying retina, their timing and frequency did not allow them to capture the dynamic changes visualized in our study.^32^–^34^

In summary, our study characterized the rabbit eye model for the implantation of subretinal prosthetic devices designed for the human eye. The rabbit eye model has a time window of 2 days postoperatively for evaluation due to the nature of the rabbit's merangiotic retina. With the surgical technique described, human-sized retinal implants can be implanted into rabbit eyes with an 85% success rate. With this surgical technique and eye model, our previous work demonstrated that the recorded visual electrophysiology signals were quantitatively responsive to the engineered stimulations and provided biological feedback for further engineering optimization.^22^ This surgical technique improves the usefulness of the rabbit eye for the development of human retinal implants. Otherwise, this easy to handle and inexpensive experimental animal would not be quite as useful in this area of research.

## Supplementary Material

Supplement 1
